# Guided imagery relaxation therapy on preoperative anxiety: why did the authors omit pain data?

**DOI:** 10.1590/1518-8345.4716.3382

**Published:** 2020-10-05

**Authors:** Richard Gray, Ellie Brown

**Affiliations:** 1 La Trobe University, College of Science Health and Engineering, Melbourne, Victoria, Australia.; 2 University of Melbourne, Centre for Youth Mental Health, Melbourne, Victoria, Australia.

**Letter to the Editor regarding the article “Felix MMS, Ferreira MBG, Oliveira LF, Barichello E, Pires PS, Barbosa MH. Guided imagery relaxation therapy on preoperative anxiety: a randomized clinical trial. Rev. Latino-Am. Enfermagem. 2018;26:e3101. DOI: http://dx.doi.org/10.1590/1518-8345.2850.3101.”**

Dear editors, we read with interest the triple-blind trial of guided imagery relaxation on preoperative anxiety by Felix^( 1 )^ and published in Revista Latino-Americana de Enfermagem.

The authors recruited 24 patients receiving bariatric surgery that were randomised to receive standard of care plus 20 minutes of guided imagery relaxation (n = 12) or standard care alone (n = 12). Two outcomes were reported in the published manuscript: state anxiety and cortisol level. The authors concluded that guided imagery relaxation is effective at reducing state anxiety and blood cortisol in the preoperative period^( 1 )^. The trial was prospectively registered with the Brazilian Clinical Trials Registry (RBR-5qywrf). According to the registry entry, the primary outcomes of the trial were postoperative pain and cortisol. Anxiety is listed as a secondary outcome. As far as we can determine, the pain data have not been reported in the manuscript. One of the primary reasons trials need to be registered is to ensure that authors do not selectively report or omit outcomes that may misrepresent the effectiveness of an experimental intervention^( 2 )^. The authors must make the pain data available and justify why these data were initially omitted from the manuscript. As part of the peer-review process reviewers and editors should have reconciled the trial registry entry with the submitted manuscript; it would be informative if the editors could confirm this was done. If this was done can the editors, then explain why the authors were not challenged about the omission of pain data. Alternatively, if the manuscript and registry entry were not checked the editors may need to consider if there has been a failure of the Journal’s editorial and peer review processes.

In our opinion, the omission of the pain data means that the reporting of the trial by Felix^( 1 )^ is incomplete and inconsistent with the trial registry entry. Consequently, the reported conclusions are unsound. We look forward to a response from both the study authors and journal editors.
