# Supplementary material for: Guided imagery relaxation therapy on preoperative anxiety: why did the authors omit pain data?
Source: Rev Lat Am Enfermagem. 2020 Oct 5;28:e3382. doi: 10.1590/1518-8345.4716.3382 (PMC7538060; doi:10.1590/1518-8345.4716.3382)
Supplement: Related texts

# Reply: Response of the authors of the article “Felix MMS, Ferreira MBG, Oliveira LF, Barichello E, Pires PS, Barbosa MH. Guided imagery relaxation therapy on preoperative anxiety: a randomized clinical trial. Rev. Latino-Am. Enfermagem. 2018;26:e3101. DOI: http://dx.doi.org/10.1590/1518-8345.2850.3101.”

**Authors:** Márcia Marques dos Santos Felix, Maria Beatriz Guimarães Ferreira, Lucas Felix de Oliveira, Elizabeth Barichello, Patricia da Silva Pires, Maria Helena Barbosa1

**Affiliations:** 1 Universidade Federal do Triângulo Mineiro, Departamento Didático Científico de Enfermagem na Assistência Hospitalar, Uberaba, MG, Brazil.; 2 Prefeitura Municipal de Uberaba, Secretaria de Saúde, Uberaba, MG, Brazil.; 3 Universidade Federal da Bahia, Instituto Multidisciplinar em Saúde, Vitória da Conquista, BA, Brazil.

The question sent to the journal’s Editor about the published article is mainly based on the statement that the primary outcome of the clinical study record was the assessment of pain intensity, and that the data referring to pain is not mentioned in the published article. Anxiety and blood cortisol levels are described as a secondary outcome.

As noted in the title of the published article: “Image-guided relaxation therapy on preoperative anxiety: a randomized clinical trial” and in the objective described “ *to evaluate the effect of image-guided relaxation therapy on state-anxiety and cortisol in the immediate preoperative period in patients undergoing bariatric surgery by videolaparoscopy* ”, this article intended to present the results related to these variables: anxiety and cortisol. It should be noted that at the end of the manuscript there is a description that the article was extracted from the PhD thesis entitled: “Image-guided relaxation therapy on anxiety and pain in patients who underwent bariatric surgery: a randomized clinical trial”, presented to the Federal University of Triângulo Mineiro, Uberaba, MG, Brazil; therefore, it is the publication of part of the results obtained in the thesis.

The pain outcome was not described in the objective of the article as it will be described in another publication, which the authors have been preparing, which does not mean an intentional omission on the part of the authors, but a decision to publish the results independently in a specialized journal.

The statement described by the contestant that the omission of this data makes the publication inconsistent with the registration of the clinical trial does not proceed, since the outcome was presented in the thesis; however, at the time of submission of the publication, it was decided to treat the results regarding pain intensity in another submission.

Sincerely,

The authors.
